# Tricuspid flow and regurgitation in congenital heart disease and pulmonary hypertension: comparison of 4D flow cardiovascular magnetic resonance and echocardiography

**DOI:** 10.1186/s12968-017-0426-7

**Published:** 2018-01-15

**Authors:** Mieke M. P. Driessen, Marjolijn A. Schings, Gertjan Tj Sieswerda, Pieter A. Doevendans, Erik H. Hulzebos, Marco C. Post, Repke J. Snijder, Jos J. M. Westenberg, Arie P. J. van Dijk, Folkert J. Meijboom, Tim Leiner

**Affiliations:** 1 Department of Cardiology, University Medical Centre Utrecht, Utrecht, the Netherlands; 2 ΙCΙN-Netherlands Heart Institute, Utrecht, the Netherlands; 3 Department of Radiology, University Medical Centre Utrecht, Heidelberglaan 100, 3584 CX Utrecht, the Netherlands; 4 Department of Paediatric Physical Therapy and Exercise Physiology, Child Development and Exercise Centre, Wilhelmina Children’s Hospital, University Medical Centre Utrecht, Utrecht, the Netherlands; 5 Department of Cardiology, Antonius Hospital, Nieuwegein, the Netherlands; 6 Department of Pulmonology, Antonius Hospital, Nieuwegein, the Netherlands; 7 Department of Cardiology, Radboud University Medical Centre Nijmegen, Nijmegen, the Netherlands; 8 Department of Radiology, Leiden University Medical Center, Leiden, The Netherlands

**Keywords:** Tricuspid regurgitation, 4D-flow MRI, Echocardiography, Congenital heart disease, Pulmonary hypertension

## Abstract

**Background:**

Tricuspid valve (TV) regurgitation (TR) is a common complication of pulmonary hypertension and right-sided congenital heart disease, associated with increased morbidity and mortality. Estimation of TR severity by echocardiography and conventional cardiovasvular magnetic resonance (CMR) is not well validated and has high variability. 4D velocity-encoded (4D-flow) CMR was used to measure tricuspid flow in patients with complex right ventricular (RV) geometry and varying degrees of TR. The aims of the present study were: 1) to assess accuracy of 4D-flow CMR across the TV by comparing 4D-flow CMR derived TV effective flow to 2D-flow derived effective flow across the pulmonary valve (PV); 2) to assess TV 4D-flow CMR reproducibility, and 3) to compare TR grade by 4D-flow CMR to TR grade by echocardiography.

**Methods:**

TR was assessed by both 4D-flow CMR and echocardiography in 21 healthy subjects (41.2 ± 10.5 yrs., female 7 (33%)) and 67 RV pressure-load patients (42.7 ± 17.0 yrs., female 32 (48%)). The CMR protocol included 4D-flow CMR measurement across the TV, 2D-flow measurement across the PV and conventional planimetric measurements. TR grading on echocardiographic images was performed based on the international recommendations. Bland-Altman analysis and intra-class correlation coefficients (ICC) were used to asses correlations and agreement.

**Results:**

TV effective flow measured by 4D-flow CMR showed good correlation and agreement with PV effective flow measured by 2D-flow CMR with ICC = 0.899 (*p* < 0.001) and mean difference of −1.79 ml [limits of agreement −20.39 to 16.81] (*p* = 0.084). Intra-observer agreement for effective flow (ICC = 0.981; mean difference − 1.51 ml [−12.88 to 9.86]) and regurgitant fraction (ICC = 0.910; mean difference 1.08% [−7.90; 10.06]) was good. Inter-observer agreement for effective flow (ICC = 0.935; mean difference 2.12 ml [−15.24 to 19.48]) and regurgitant fraction (ICC = 0.968; mean difference 1.10% [−7.96 to 5.76]) were comparable. In 25/65 (38.5%) TR grade differed by at least 1 grade using 4D-flow CMR compared to echocardiography.

**Conclusion:**

TV effective flow derived from 4D-flow CMR showed excellent correlation to PV effective flow derived from 2D-flow CMR, and was reproducible to measure TV flow and regurgitation. Twenty-five out of 65 patients (38.5%) were classified differently by at least one TR grade using 4D-flow CMR compared to echocardiography.

## Background

Tricuspid valve (TV) regurgitation (TR) frequently complicates pulmonary hypertension (PH) and congenital heart defects (CHD) associated with right ventricular (RV) pressure or volume overload. The presence and severity of TR in these patient groups is independently associated with both increased morbidity and increased mortality [1–3].

The complex structure of the valve and the degree of motion throughout the cardiac cycle make assessment of TR severity difficult. When the right ventricle (RV) dilates, RV geometry is altered, making valve structure and flow patterns even more difficult to evaluate [4]. Severity of TR is primarily assessed using qualitative and semi-quantitative (color) Doppler echocardiography [5, 6]. However, these measurements are less well validated for TR than for other valvular regurgitations and have high inter-observer variability [5, 7, 8]. Consequently, if only echocardiography is used, there is substantial risk of misjudging severity of TR [8], influencing clinical decision-making.

Cardiovascular magnetic resonance imaging (CMR), using 2D velocity-encoded imaging (2D-flow), is the reference standard for assessment and follow-up of aortic and pulmonary regurgitation in patients with CHD [9]. However, for both atrioventricular valves, direct flow measurement with 2D-flow CMR is regarded as less reliable [9, 10], mostly due to the large degree of valvular through-plane motion [11, 12]. Alternatively, indirect quantification – combining planimetric RV stroke volume and direct flow measurement across the pulmonary valve (PV) – is used, but this approach also introduces multiple sources of error [12, 13].

Four-dimensional velocity-encoded (4D-flow) CMR encodes velocity simultaneously in three orthogonal directions, thereby enabling direct flow measurement, perpendicular to the annular plane, throughout the entire cardiac cycle [10, 14]. Tricuspid flow measurement by 4D-flow CMR has been validated in patients with normal RVs, as well as in patients with tetralogy of Fallot without TR [14, 15]. However, to the best of our knowledge, a validation study in patients with complex RV geometry and varying degrees of TR has not been done. Furthermore, it is unknown to what extent TR grade by TV 4D-flow CMR differs from the current clinical standard for assessing TR; echocardiography.

The aims of the present study were: 1) to correlate effective flow across the TV measured by 4D-flow CMR to 2D-flow derived effective flow across the *PV,* in patients with complex RV geometry; 2) to assess reproducibility of 4D-flow measurement across the TV, and 3) to compare TR grade by 4D-flow CMR to TR grade by echocardiography.

## Methods

A prospective, cross-sectional study was performed in three tertiary referral hospitals including healthy subjects (*n* = 21), patients with pulmonary hypertension (PH; *n* = 30), patients with isolated valvular pulmonary stenosis (PS) or tetralogy of Fallot (*n* = 21) and systemic RV patients (*n* = 16). CMR and transthoracic echocardiography were performed at a single center by dedicated and experienced staff.

### In- and exclusion criteria

The PH group comprised only patients with pre-capillary pulmonary hypertension – either idiopathic pulmonary arterial hypertension or chronic thromboembolic pulmonary hypertension. In all PH patients, the diagnosis of pre-capillary PH had been previously confirmed by right heart catheterization. Pre-capillary PH was defined as mean pulmonary artery pressure of ≥ 25 mmHg and a pulmonary capillary wedge pressure of ≤ 15 mmHg [16]. All PH patients were on PH-specific therapy when entering the study. The CHD cohort consisted of two major groups: 1) patients with a systemic RV either after atrial switch procedure for transposition of the great arteries or congenitally corrected transposition of the great arteries, and 2) patients with isolated valvular pulmonary stenosis (PS) or tetralogy of Fallot with pulmonary valvular or homograft stenosis. Patients with contra-indications for CMR were excluded. A group of healthy subjects between 18 and 60 years old served as control population. Subjects were screened using physical examination, medical history and electrocardiogram and excluded if these investigations or subsequent CMR and echocardiogram showed any abnormalities.

The study protocol conformed to the ethical guidelines of the 1975 Declaration of Helsinki. The medical ethics committees of all participating centers approved the study and written informed consent was obtained from all participants prior to inclusion.

### General patient data

Demographic data, electrocardiogram (ECG), basic echocardiographic measurements and functional capacity were obtained for each patient. Echocardiography and CMR were performed consecutively with less than 1 h in between investigations. Patients and controls did not use medication between investigations, nor did they perform any strenuous physical activity.

### Cardiovascular magnetic resonance imaging

#### Cine imaging, 2D flow imaging & functional analysis

All participants were imaged using a pre-defined imaging protocol without sedation. A commercially available 1.5-T CMR system (Ingenia R4.1.2; Philips Healthcare, Best, the Netherlands) was used, with a dedicated chest phased-array parallel-imaging capable surface coil. Balanced steady-state free precession cine images were acquired in various orientations during repeated end-expiratory breath holds. Multi-slice cine short-axis acquisitions were acquired from the apex up to and including the atrioventricular valves and the entire left ventricle and RV. The following sequence parameters were used: TR/TE 3.4/1.69 ms, voxel size 1.3 × 1.3 × 8.0 mm, flip angle: 55^o^ and a temporal resolution of 30 phases per cardiac cycle. Effective flow was measured at the level of the pulmonary valve - or aortic valve in case of systemic RV – using 2D-flow CMR with a retrospectively ECG-gated, velocity-encoded phase-contrast sequence (TR/TE 5.2/3.1 ms, voxel size 2.5 × 2.5x8mm, flip angle 12^o^, field of view 320, matrix 128 × 100, 20 phases per cardiac cycle). The VENC was set to 150 cm/s, in case of a PS it was individually adapted to yield images without aliasing artifacts.

RV volumetric analysis was performed as previously described, by manual tracing of endocardial and epicardial contours in end-diastolic and end-systolic phase in all slices, using Qmass MR Research edition (version 7.4, Medis, Leiden, The Netherlands), excluding the trabeculae and papillary muscles from the ventricular blood pool volume [17].

The following parameters were determined for the RV: end-diastolic volume (EDV), end-systolic volume (ESV), stroke volume (SV), ejection fraction (EF) and RV mass. Quantification of 2D flow across the pulmonary valve (or aortic valve in systemic RVs) was performed using Medis Qflow (version 5.5, Medis, Leiden, the Netherlands) and used to calculate the effective flow across the pulmonary valve (2D-flow PV). All volumetric data were indexed for body surface area. At end-diastole the maximum tricuspid annular diameters were measured in cine RV 2-chamber and 4-chamber views.

### 4-dimensional velocity encoded CMR

For planning purposes 4-chamber, 2-chamber (of both ventricles) and perpendicular views of each of the four cardiac valves were used – to ensure that the 4D-flow volume would enclose all valves during diastole and systole. Velocity data were acquired in three orthogonal directions. The 4D-flow CMR acquisition was based on the protocol previously described by Westenberg et al. [14]. The following acquisition parameters were used: TR/TE 7.3/3.9 msec, respectively; field of view 370 × 219 × 63 mm; 3D volume imaging with 63-mm slab thickness reconstructed into 28 3.5 mm slices; 10° flip angle; acquisition voxel 3.43 × 3.65 × 3.5 mm; reconstructed voxel size 2.9 × 2.9 × 3.5 mm; 1 signal acquired; retrospective gating with 20-30% acceptance window, with 30 phases reconstructed during 1 average cardiac cycle; maximal velocity encoding of 150 cm/s in all three directions. To reduce acquisition time, echo planar imaging was used with a factor of 5.

**Fig. 1:**
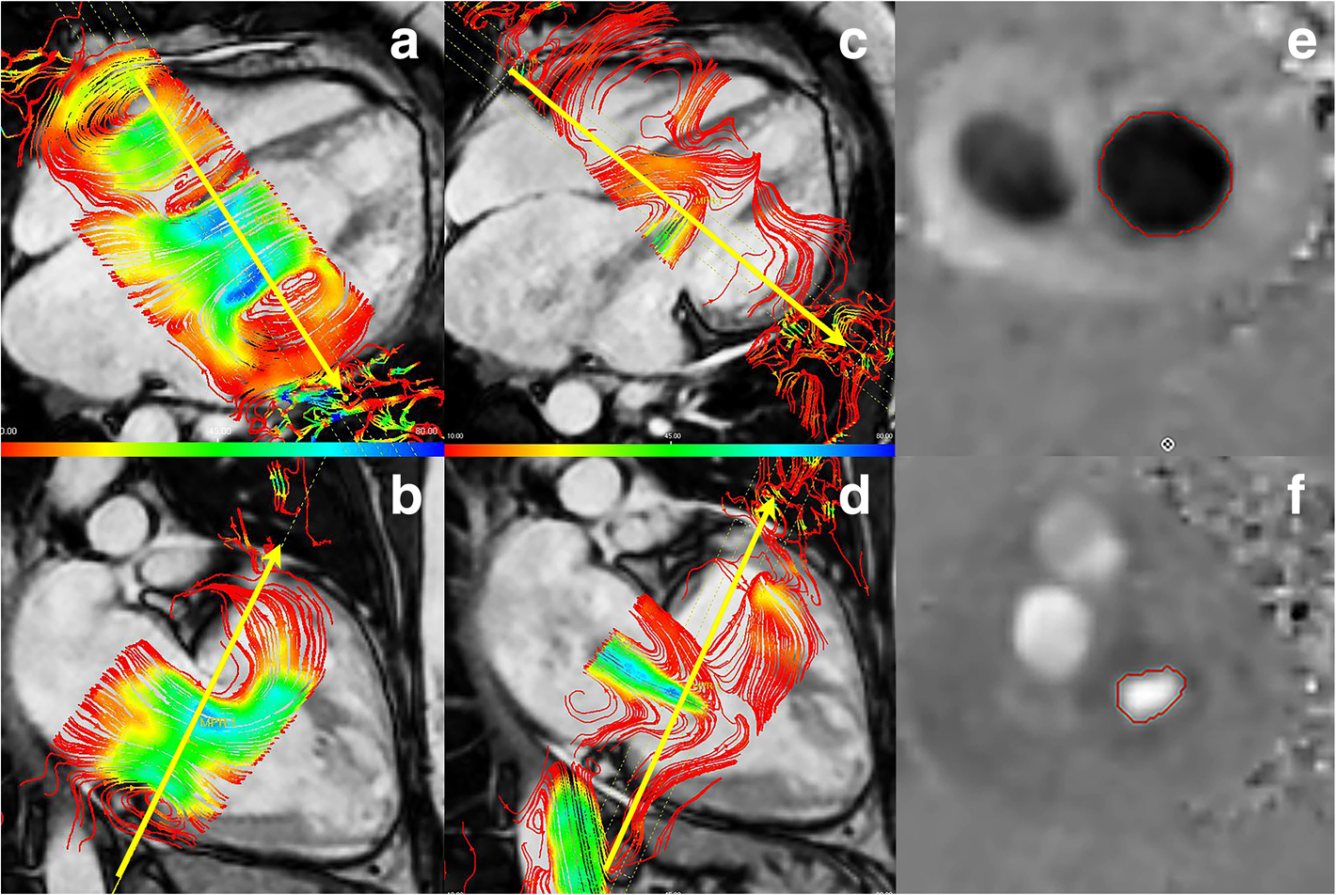
Example of 4D flow. Example of 4D-flow CMR analysis. In **a**-**d** the annular plane is reconstructed perpendicular to the flow direction, in figure **e** + **f** the flow contours are drawn in the reconstructed through-plane flow slice

Analysis of through-plane flow across the TV was performed with Mass (version 5.1, Medis) In short, the TV plane was reconstructed in each cardiac phase using two orthogonal planes, perpendicular to the flow across the TV (Fig. 1 a-d). After the valve plane was reconstructed for each phase of the cardiac cycle, the through-plane flow was reformatted in five parallel planes with a slice gap of 5 mm. Subsequently, contours were drawn outlining the flow of the valve of interest (Fig. 1-e&f) for each phase. Through-plane motion correction using the velocity of myocardium was taken into account by a indicating a region of interest in the myocardium. Finally, forward and backward flow, regurgitant fraction and effective flow were derived from 4D-flow CMR. To illustrate the impact of measurement plane reconstruction perpendicular to the flow direction in 4D-flow CMR, we performed flow analysis using a static tricuspid valvular plane in 15 random datasets (i.e. in essence comparable to 2D-flow across the TV).

To assess the accuracy of the 4D flow measurement across the TV, 4D-flow derived TV effective flow was compared to the current reference: 2D-flow derived pulmonary effective flow. Severity of TR was classified according to regurgitation fraction: 0-10% (absent/trace); 10-20% (mild); 20-40% (moderate); > 40% (severe) [18].

### Echocardiography

Echocardiography was performed using a Toshiba Artida system (Toshiba, Tokyo, Japan) with a 5-MHz transducer. Analysis was performed offline in XCelera (version 4.1.1.1133 - 2013; Philips Healthcare). RV systolic pressure (RVSP) was calculated using the Bernoulli equation with maximum velocity of TR or by maximal pulmonary valve gradient (only in PS patients with valvular stenosis), plus estimated right atrial pressure. In systemic RV patients the systemic systolic blood pressure at rest was used as RVSP. Tricuspid annular plane systolic excursion (TAPSE) and systolic velocity using tissue (TDI S′) Doppler imaging were measured in 4-chamber view.

TR was visualized in a parasternal 2-chamber RV, parasternal short axis aorta orientation, subcostal and in the modified apical 4-chamber view – if possible an apical 2-chamber-RV view was also used. To quantify TR the following parameters were taken into account: vena contracta, early tricuspid inflow velocity, hepatic vein systolic flow reversal, color Doppler flow jet area and density of TR Doppler signal [19]. The severity of TR was graded none/trace, mild, moderate or severe based on these (semi-) quantitative and qualitative assessment - by an experienced imaging-cardiologist (F.M.) blinded to 4D-flow CMR results.

#### Reproducibility

To assess intra-observer reproducibility 15 patients were re-analyzed by the first observer (CMR - M.S.; echocardiography – F.M.). Inter-observer reproducibility was assessed in the same 15 patients by a second observer (CMR - M.D.; echocardiography – G.S.). Both observers were blinded for the previous results and the first and second analysis were at least 1 month apart.

#### Statistical analysis

For all continuous variables, the distribution was tested using the Shapiro-Wilk test and by plotting histograms. Continuous data were expressed as mean value ± standard deviation (SD) or median [range] as appropriate. Continuous data were compared between groups using ANOVA with posthoc Dunnet’s test or the Kruskal Wallis analysis of variance, depending on distribution of data and residuals. Categorical data was presented as absolute number followed by percentage, the agreement of TV regurgitation grade by echocardiography and 4D-flow CMR and reproducibility measurements for echocardiography, was assessed using linear weighted Kappa (< 0.2 slight, 0.2-0.4 fair, 0.4-0.6 moderate, 0.6-0.8 considerable and >0.80 almost perfect agreement).

Agreement between 2D-flow derived PV effective stroke volume and 4D-flow CMR derived TV effective SV as well as between repeated measures was assessed using intraclass correlation coefficients (ICC) and paired-samples T-test. Furthermore, Bland Altman plots were constructed. All data analyses were performed in SPSS statistics (version 20.0, International Business Machines, Inc., Chicago, Illinois, USA). *P*-values of <0.05 were considered statistically significant.

## Results

### Demographic data

All demographic and baseline data are listed in Table 1. In short, PH patients were older than the control group and CHD patients. Patients with systemic RVs had the highest RVSP. Basic volumetric measurements are also listed in Table 1; RV volumes were increased in systemic RV and PH patients compared to controls.

**Table 1:** Demographic data

	Controls (*n* = 21)	CHD (*n* = 37)		PH (*n* = 30)	*p*-value
		PS (*n* = 21)	SystRV (*n* = 16)		
Age (yrs)	41.2 ± 10.5	30.6 ± 12.6^#^	36.6 ± 8.4	54.4 ± 15.6^**^	<0.001
BSA (m^2^)	1.93 ± 0.21	1.90 ± 0.15	1.94 ± 0.15	1.92 ± 0.26	0.97
RVSP (mmHg)	–	50 [40-111]	108 [89-127]	54 [23-100]	0.025
VO_2_/kg (ml/min)	–	29.7 ± 6.6	27.1 ± 6.6	17.0 ± 4.1	<0.001
%PredVO_2_/kg	–	83.8 ± 17.2	83.0 ± 18.5	62.9 ± 12.9	<0.001
QRS (msec)	93.1 ± 13.7	133.1 ± 34.4^**^	107.2 ± 16.2	97.5 ± 24.2	<0.001
Heart rate	61 ± 9	69 ± 9	59 ± 8	69 ± 12	0.013
RVEDV (ml/m^2^)	97 [60-134]	95 [71-170]	109 [73-222]	110 [52-343]	0.48
RVESV (ml/m^2^)	46 [20-59]	49 [26-122]	58 [26-170] ^*^	60 [16-271]^*^	0.009
RVEF (%)	54.8 ± 4.6	49.0 ± 9.2	45.5 ± 10.7^#^	41.1 ± 11.4^**^	0.017

General demographic data for all groups. To test for differences between the different groups (last column) ANOVA with posthoc Dunnets (controls as reference) was used for normally distributed data and Kruskal Wallis analysis of variance with Mann Whitney U tests for non-normally distributed data. ^#^*p* < 0.05, *p < 0.01, **p < 0.001. *Abbreviations:* *BSA* body surface area, *RVSP* right ventricular systolic pressure, *VO*_*2*_*/kg* peak oxygen uptake per kg, *%PredVO*_*2*_*/kgg* % of predicted peak oxygen uptake, *RV* right ventricular, *EDV* end-diastolic volume *ESV* end-systolic volume, *EF* ejection fraction

4D-flow and 2D-flow CMR measurements are depicted in Table 2. Acquisition time for the 4D-flow CMR dataset was 3.5-7 min; acquisition time of cine CMR images necessary for post-processing was approximately 7 min. Lastly, acquisition time for echocardiographic images related to assessment of TR was approximately 15 min. Analysis time for 4D-flow CMR across the TV was approximately 35 min per patient; analysis with a static plane (2D TV flow) was approximately 10 min. Analysis of TR grade on echocardiography took on average 6 min per patient.

**Table 2:** CMR measurements

	Controls (*n* = 21)	CHD (*n* = 37)		PH (*n* = 30)	
		PS (*n* = 21)	Syst RV (*n* = 16)		*p*-value
4D-TV FW (ml)	111.5 ± 27.3	91.2 ± 15.2^#^	95.4 ± 19.8	87.2 ± 21.7^**^	0.23
4D-TV BW (ml)	8.3 [2.0-24.1]	8.4 [5.1-25.9]	10.7 [3.7-34.0]	7.3 [2.2-57.7]	0.61
4D-SV TV (ml/m^2^)	52.4 ± 9.4	42.5 ± 5.7^**^	41.7 ± 8.0^**^	38.7 ± 9.6^**^	0.13
4D-TV reg (%)	7.9 [1.9-17.6]	9.3[5.4-25.0]	12.5 [5.1-40.1]	9.2 [3.2-49.6]	0.307
2D-SV PA (ml/m^2^)	49.5 ± 7.7	42.1 ± 4.8^*^	42.8 ± 7.5^#^	38.0 ± 9.6^**^	<0.001
TVann 4CH (mm)	37.4 ± 4.1	38.8 ± 5.8	42.3 ± 5.0^*^	39.6 ± 4.4	0.032
TVann 2CH (mm)	37.5 ± 4.3	37.7 ± 5.3	38.5 ± 4.5	38.5 ± 3.6	0.84

Results of volumetric and 4D flow CMR

To test for differences between the different groups (last column) ANOVA with posthoc Dunnets (controls as reference) was used for normally distributed data and Kruskal Wallis analysis of variance with Mann Whitney U tests (controls as reference) for non-normally distributed data. ^#^*p* < 0.05, *p < 0.01 and **p < 0.001. *Abbreviations:* *4D-TV FW* 4D tricuspid valve forward flow, *4D-TV BW* 4D tricuspid valve backward flow, *4D-SV TV* 4D tricuspid valve effective flow, *4D-TV reg* 4D flow tricuspid valve regurgitation, *2D-SV PA* 2D pulmonary valve effective flow, *TVann 4CH* tricuspid annulus diameter in 4-chamber view, *TVann 2CH* tricuspid valve annulus diameter in 2-chamber view

### Accuracy of 4D-SV TV compared to reference 2D-SV PV

**Fig. 2:**
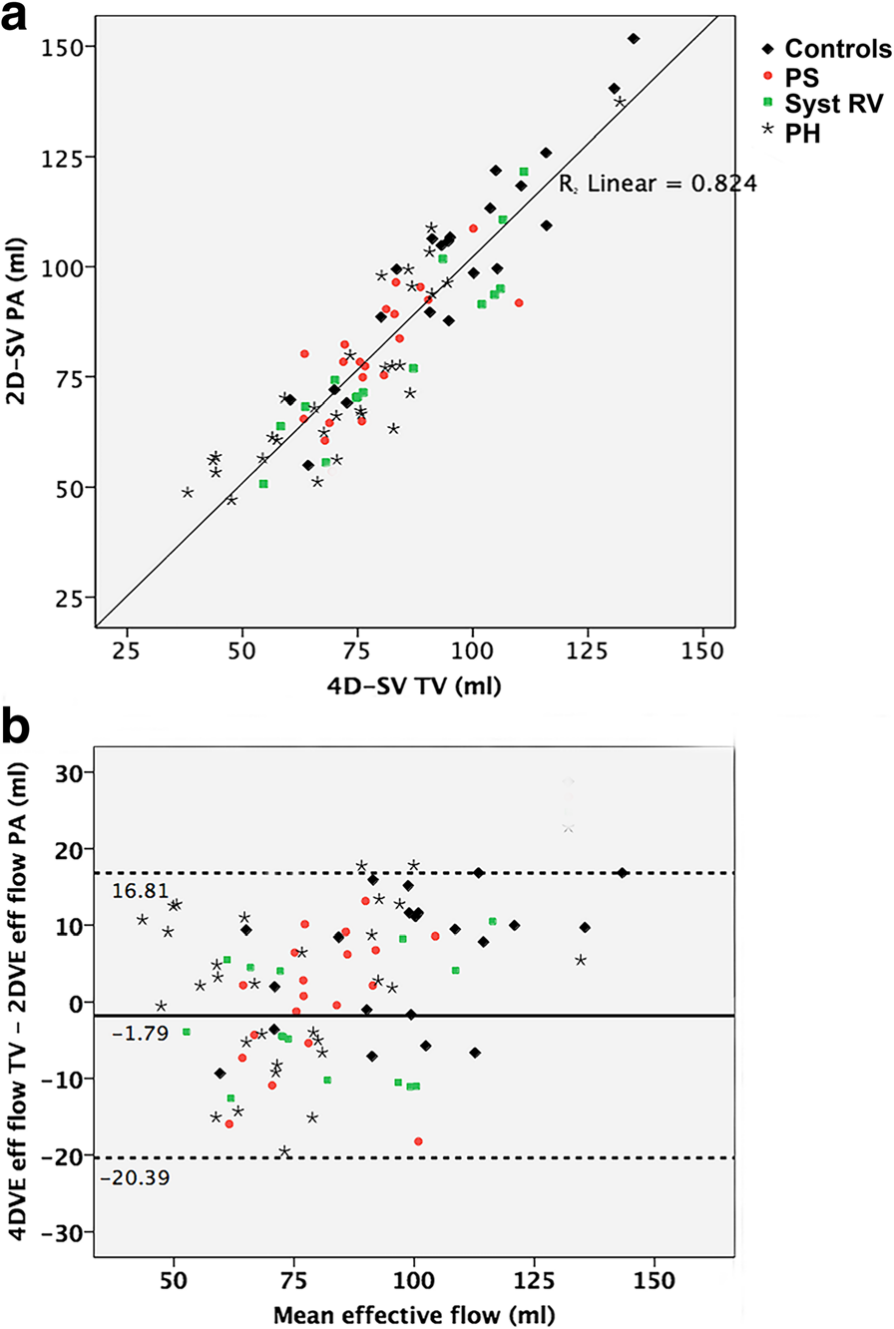
Accuracy of 4D-flow CMR. **a**) depicts the correlation between effective flow across the tricuspid valve using 4D-flow CMR (4D-flow TV) and effective flow across the pulmonary valve using 2D-flow CMR (2D-flow PV). **b**) depicts a Bland-Altman analysis with the difference between both effective flow measurements on the x-axis and the average of both measurements on the y-axis

TV 4D-flow CMR analysis was possible in 67/67 patients (100%). Effective flow measured across the TV by 4D-flow CMR could be compared to 2D-flow derived effective flow across the PV in 85/88 subjects, due to poor quality of 2D-flow images in three subjects (Fig. 2a-c). The intra-class correlation coefficient between both measurements was 0.90 (95% confidence interval 0.85-0.94; *p* < 0.001) and the R^2^ was 0.83. Mean difference in 4D-flow derived TV effective flow vs 2D-flow derived PV effective flow was −1.6 ml (*p* = 0.083) with limits of agreement: −20.0 to 16.8 ml.

**Fig. 3:**
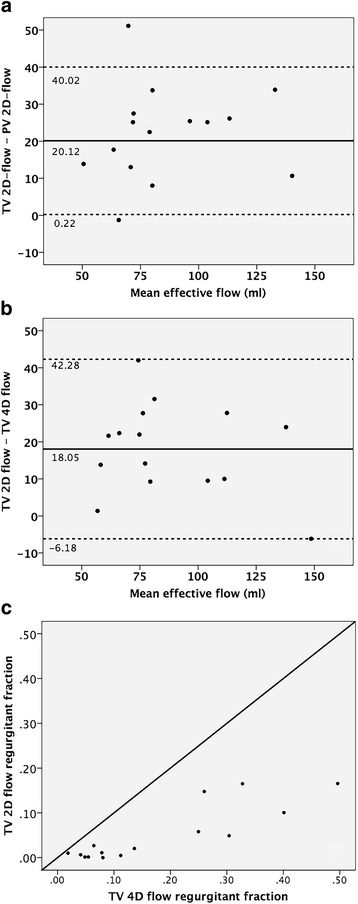
Tricuspid valve 2D-flow versus 4D-flow. Bland-Altman plots of tricuspid valve effective flow using a static annular plane (similar to 2D flow) versus effective flow across the pulmonary valve (**a**) and versus 4D flow (**b**). **c**) depicts tricuspid regurgitant fraction using a static annular plane (2D flow on y-axis) versus 4D flow (x-axis)

Analysis of TV effective flow, using a static annular plane – similar to TV 2D flow method – was performed in 15 datasets. Intra-class correlation coefficients for TV 2D-effective flow vs. PV 2D-effective flow, TV 2D-effective flow vs. TV 4D-effective flow and TV regurgitant fraction using 2D flow vs. 4D-flow were respectively 0.666 (p < 0.001), 0.767 (*p* < 0.001) and 0.389 (*p* = 0.002). Effective flow was significantly overestimated by TV 2D-flow compared to both other methods (*p* < 0.001; Fig. 3), regurgitant fraction was significantly underestimated (*p* < 0.001; Fig. 3).

### Reproducibility

**Fig. 4:**
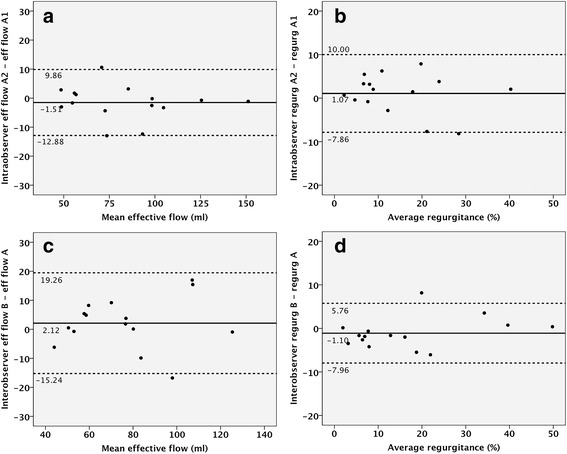
Reproducibility of 4D flow CMR measurements. **a**&**b** Bland-Altman analysis of intra-observer repeated measurements of effective flow (**a**) and regurgitation (**b**). **c**&**d** Bland-Altman analysis of inter-observer repeated measurements of effective flow (**c**) and regurgitation (**d**)

Reproducibility measurements are listed in Table 3 and are shown in Fig. 4. Both intra- and interobserver measurements yielded good intra-class correlation coefficients (all > 0.91 and *p* < 0.001) for TV 4D-flow CMR. Mean intra- and interobserver differences in forward flow and effective flow were small with good limits of agreement (Table 3). The mean difference and limits of agreement for measurement of TR (%) were acceptable for both intra- and inter-observer measurements (1.08% [−7.90; 10.06] and −1.10% [−7.96; 5.76], respectively).

**Table 3:** Intra- and interobserver agreement

	Mean Δ	*p*-value^1^	ICC	*p*-value^2^
Intraobserver				
Forward flow (ml)	1.08 ± 4.58	0.911	0.963	<0.001
Stroke volume (ml)	−1.51 ± 5.80	0.329	0.981	<0.001
Regurgitance (%)	−1.07 ± 4.58	0.378	0.910	<0.001
Interobserver				
Forward flow (ml)	1.44 ± 9.26	0.370	0.911	<0.001
Stroke volume (ml)	2.12 ± 8.86	0.556	0.935	<0.001
Regurgitance (%)	−1.1 ± 3.5	0.242	0.968	<0.001

Mean difference (Δ) between repeated measures and significance were tested with a paired Student T-test and agreement using intra-class correlation coefficient (ICC)

^1^*p*-value using paired Student's T test

^2^*p*-value for intra-class correlation coefficient (ICC)

Reproducibility of echocardiography showed a kappa value of 0.57 (95% CI 0.30-0.84) for intra-observer agreement and 0.66 (95% CI 0.39-0.92) for inter-observer agreement. During second observation TR grade was classified differently in 6/15 (= 40%) of patients by the same observer, and in 5/15 (= 33%) by a second observer.

### TR grade by 4D-flow CMR vs echocardiography

Severity of TR could be graded by 4D-flow CMR in all 67 patients, and in 65/67 (97%) patients by echocardiography. Of these patients, 40/65 patients (61.5%) showed consistent results for echo and 4D-flow CMR, but 25/65 (38.5%) were classified differently by at least 1 grade using 4D-flow CMR compared to echocardiography (Table 4). Both methods showed only moderate agreement; with a linear weighted kappa of 0.52 (95%-confidence interval 0.37-0.67).

**Table 4:**
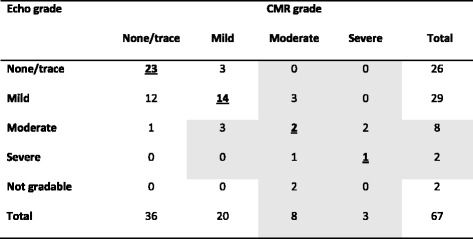
Echocardiography vs 4D-flow CMR

Contingency table depicting the results for TR grading by echocardiography versus 4D-flow CMR (clinically most relevant group, with either moderate or severe TR, highlighted). The linear weighted kappa for agreement between both methods was 0.52 (95%-confidence interval 0.37-0.67)

A trace to mild TR grade by echocardiography excludes moderate or severe TV regurgitation by 4D-flow CMR in 95% of patients (=negative predictive value). However, moderate or severe TR grade by echocardiography corresponded to a moderate or severe TR grade by 4D-flow CMR in only 60% of patients (=positive predictive value). Of the 14 patients with moderate or severe TR on echocardiography or 4D-flow CMR, 2 could not be classified by echocardiography and 9/12 (75%) were classified differently by both methods.

## Discussion

In this study, we assessed the feasibility, accuracy and reproducibility of 4D-flow CMR derived TV flow in patients with RV heart disease and varying degrees of TV regurgitation. Four-dimensional-flow derived TV flow proved to be feasible in all patients and correlated well to our reference standard 2D-flow derived PV effective flow. Furthermore, reproducibility of flow volumes and regurgitation by 4D-flow CMR were good, even in this complex patient population. In 25/65 (38.5%) of the patients, 4D-flow CMR led to a different grading of TR compared to echocardiography.

### 4D-flow CMR of the tricuspid valve

In our patient cohort, effective flow across the TV measured with 4D-flow CMR showed excellent correlation to effective flow across the PV measured with 2D-flow CMR. This is true for both patient groups (ICC 0.870) as well as healthy controls (ICC 0.895). Conversely, TV flow measured without reconstruction of a measurement plane perpendicular to the flow (similar to 2D flow CMR), significantly overestimated effective flow volume compared to the PV effective flow. Our results are in line with previous reports on 4D-flow CMR derived TV flow in patients without RV heart disease, by Westenberg et al., and those with tetralogy of Fallot without TR, by van der Hulst et al. [14, 15]. The limits of agreement for effective flow (i.e. stroke volume) between TV 4D-flow CMR and PV 2D-flow CMR varied between −20.0 to +16.8 ml. These values are acceptable, as even for repeated 2D-flow measurements of semi-lunar valves there is considerable inter-observer and interscan variation [20, 21]. For example, Kondo et al. reported a relative difference of 7.0 ± 5.6% for repeated PV 2D-flow CMR measurements [21]. The difference between 4D-flow CMR derived TV flow and 2D-flow CMR derived PV flow can further be explained by several other factors: 1) 2D-flow suffers from some through plane movement; 2) 2D-flow across the PV was obtained during end-expiratory breath-hold while 4D-flow CMR was obtained during free-breathing and 3) interscan variability (although time between acquisitions was < 10 min).

Thus far, reproducibility measurements have been mostly obtained in patients with structurally normal hearts or focused on other applications of 4D-flow imaging (i.e. peak flow velocity and wall stress) [14, 22]. In patients with RV dilation and hypertrophy, TV geometry is distorted [4]. Hence, reproducibility of 4D-flow CMR derived TV flow in this population cannot be extrapolated from healthy subjects or patients with structurally normal hearts. The present study fills this gap and demonstrates good intra-observer and inter-observer agreement with acceptable limits of agreement and excellent ICC coefficients (all >0.90). The reproducibility in our cohort is comparable to limits of agreement reported in TV flow of healthy controls by Westenberg et al. and 2D-flow imaging of pulmonary or aortic valves [20, 21].

### Echocardiography vs 4D-flow CMR

We demonstrate a discrepancy between 4D-flow CMR and echocardiographic TR grading in our patient cohort. Twenty-five out of 65 patients (38.5%) were classified differently by at least one grade using quantitative 4D-flow CMR TR measurements compared to echocardiographic assessment. Importantly, this number was even higher in patients with severe or moderate TR (namely 9/12 = 75% of patients). With the lack of a gold standard it is difficult to be sure which of these techniques provides the most accurate results. However, TV flow assessment by 4D-flow CMR has been validated both in vitro and in vivo [10, 14, 23] and TR grading by echocardiography has shown less consistent results [5, 7, 8], which may indicate 4D-flow CMR is more reliable. Adding to this, agreement between repeated measures was very good (ICC > 0.9) for repeated 4D-flow measurements, but only moderate to considerable (Kappa 0.57 and 0.66) for repeated TR grade by echocardiography. Importantly, a trace to mild TR grade by echocardiography still excludes moderate or severe TV regurgitation by 4D-flow CMR in 95% of patients.

### Clinical implications

As is the case for many other measurements of RV size and function, echocardiography is an excellent screening tool. However, for assessment of degree of TV regurgitation, 4D-flow CMR seems of added value. If moderate or severe TR is seen on echocardiography, additional assessment of TR by means of 4D-flow CMR may help or guide clinical decision-making. In patients in whom surgery is considered, TR grading using 4D-flow CMR may improve selection of patients that potentially benefit from TV annuloplasty [24–27]. Moreover, the detailed anatomical information obtained with 4D-flow CMR (Fig. 1) may be helpful in identifying the mechanism of regurgitation and planning of intervention.

In both pulmonary hypertension patients and in CHD the degree of TR is related to symptoms and mortality [1–3, 28]. Accurate determination of the degree of TR can help physicians better understand a patients’ physiology and ensure timely referral of patients for surgery or transplant.

### Limitations

This study has limitations. First, 4D-flow CMR post processing and analysis remains time-consuming and labor-intensive (35 min), limiting its use in clinical practice for many institutions. Currently, validation of semi-automated software is underway – shortening post-processing times and making analysis less user-dependent [29]. Furthermore, in addition to observer variability, interscan variability will need to be evaluated in future studies.

The number of patients with moderate to severe TR was limited, which may limit its application in these patients.

We chose to use 2D-flow CMR derived PV flow as reference measurement for stroke volume, introducing interscan variability and through plane motion. Invasively measured stroke volumes across the pulmonary valve were not available. We did not use 4D-flow CMR for PV flow as many of our patients had severe PS with a large degree of aliasing or a homograft – for which 4D-flow CMR is not yet validated.

## Conclusion

In patients with complex RV morphology, 4-D flow CMR is a reproducible method to measure TV flow and regurgitation and effective flows showed excellent correlation to 2D-flow CMR across the PV. Twenty-five out of 65 patients (38.5%) were classified differently by at least one grade using quantitative 4D-flow CMR TR grading compared to echocardiographic assessment. However, a trace to mild TR by echocardiography excludes moderate or severe TR by 4D-flow CMR in 95% of patients. As software developments accelerate 4D-flow CMR post-processing, it can provide a reproducible method of grading TR in clinical practice and future studies.

## Abbreviations

2D-flow: Two-dimensional velocity-encoded imaging
4D-flow: Four-dimensional velocity-encoded imaging
CHD: Congenital heart defect(s)
CMR: Cardiovascular magnetic resonance
EDV: End-diastolic volume
EF: Ejection fraction
ESV: End-systolic volume
ICC: Intra-class correlation coefficient
PH: Pulmonary hypertension
PS: Pulmonary stenosis
PV: Pulmonary valve
RV: Right ventricular/right ventricle
RVSP: Right ventricular systolic pressure
SD: Standard deviation
SV: Stroke volume
TAPSE: Tricuspid annular plane systolic excursion
TDI: Tissue Doppler imaging
TR: Tricuspid regurgitation
TV: Tricuspid valve
